# Health-related quality of life and self-care in heart failure patients under telecare—insights from the randomized, prospective, controlled AMULET trial

**DOI:** 10.3389/fpubh.2024.1431778

**Published:** 2024-09-25

**Authors:** Katarzyna Piotrowicz, Paweł Krzesiński, Agata Galas, Adam Stańczyk, Janusz Siebert, Ewa Anita Jankowska, Paweł Siwołowski, Piotr Gutknecht, Piotr Murawski, Dominika Szalewska, Waldemar Banasiak, Piotr Ponikowski, Grzegorz Gielerak

**Affiliations:** ^1^Department of Cardiology and Internal Diseases, Military Institute of Medicine, Warsaw, Poland; ^2^University Center for Cardiology, Gdansk, Poland; ^3^Department of Family Medicine, Medical University of Gdańsk, Gdańsk, Poland; ^4^Department of Heart Diseases, Wroclaw Medical University, Wroclaw, Poland; ^5^Center for Heart Diseases, University Hospital, Wroclaw, Poland; ^6^Department of Cardiology, Center for Heart Diseases, 4th Military Hospital, Wroclaw, Poland; ^7^Department of Informatics, Military Institute of Medicine, Warsaw, Poland; ^8^Department and Clinic of Rehabilitation Medicine, Faculty of Health Sciences, Medical University of Gdańsk, Gdańsk, Poland; ^9^Department of Non-Surgical Clinical Sciences, Faculty of Medicine, Wroclaw University of Science and Technology, Warsaw, Poland

**Keywords:** heart failure, health-related quality of life, self-care, telecare, heart failure management

## Abstract

**Introduction:**

The growing population of heart failure (HF) patients places a burden on the healthcare system. Patient-centered outcomes such as health-related quality of life (HRQoL) and self-care behaviors are key elements of modern HF management programs. Thus, optimized strategies to improve these outcomes are sought.

**Purpose:**

To assess the effects of a new model of medical telecare on HRQoL and self-care in patients with HF (the AMULET study).

**Methods:**

The study was prospective, randomized, open-label, and controlled with two parallel groups: telecare and standard care. In the telecare group, HF nurses performed patient clinical assessments with telemedical support by a cardiologist and provided education focused on the prevention of HF exacerbation. In the standard care group, patients were followed according to standard practices in the existing healthcare system. At the baseline and at 12 months, HRQoL was assessed using the Short Form 36 (SF-36) questionnaire and the Minnesota Living with Heart Failure Questionnaire (MLwHF). The level of self-care was assessed with the 12-item standardized European Heart Failure Self-care Behavior Scale (EHFScBS-12).

**Results:**

In the overall study group, 79% of the subjects were male, the mean age was 67 ± 14 years, and 59% of the subjects were older than 65 years of age. The majority of the subjects (70%) had a left ventricular ejection fraction below 40%. After 12 months, statistically significant increases in physical component of the SF-36 (43.3 vs. 47.4 for telecare vs. 43.4 vs. 46.6 for standard care) and mental component of SF-36 (58.4 vs. 62 for telecare vs. 60.4 vs. 64.2 for standard care) were noted, with no intergroup differences. However, patients receiving telecare showed improvement in specific domains, such as physical functioning, role-physical, bodily pain, vitality, social functioning, role-emotional, and mental health. There was a significant decrease in MLwHF (29 vs. 35.0; lower is better) at follow-up for both groups. Telecare patients had a statistically significant decrease in EHFScBS-12 (lower is better) at 12 months.

**Conclusion:**

AMULET outpatient telecare, which is based on nurse-led non-invasive assessments supported by specialist teleconsultations, improved the HRQoL and self-care of HF patients after an episode of acute HF.

## 1 Introduction

The complex pathophysiology, varied clinical signs and symptoms, growing incidence, and uneven course of heart failure (HF) pose challenges to health care. The estimated prevalence of HF in the general population ranges from 0.4 to 2%. In 10 years, the number of patients with HF will increase by approximately 25% (1). While remarkable progress toward the medical management of HF has been made, clinical outcomes remain unsatisfactory. HF is a leading cause of hospitalization, and more than 40% of patients are rehospitalized for HF within 6 months of discharge (2). As such, there is a pressing need for an optimized care plan for patients with HF to reduce recurrent hospitalizations.

HF symptoms, especially shortness of breath, dyspnea, and chronic fatigue, significantly influence patients’ daily functioning, both physically and mentally. The high burden of physical and social limitations places HF patients at a high risk of psychological distress, including depression, anxiety, and adjustment disorders. The current recommendations of the European Society of Cardiology emphasize the importance of good health-related quality of life (HRQoL) as one of the aims of comprehensive care for patients with HF. The patients’ perception of the disease and their involvement in shared decision-making and the treatment process may empower them to cope with their symptoms and improve their adherence to treatment plans; these, in turn, influence health outcomes (3). In this context, health-promoting behaviors, including self-care, constitute an important element of optimal HF care, profoundly impact the life expectancy of patients, and facilitate living with the disease with the fewest possible limitations.

It has been documented that in the management of chronic diseases, an educational process enriched with behavior-changing techniques and provided by well-trained medical staff can influence health outcomes (4). In HF management, patient education regarding lifestyle recommendations is crucial; however, there is still ongoing debate about who should provide it and in what manner.

The use of digital tools in the management of cardiovascular disease is rapidly growing. Telemedicine solutions for HF patients in various intervention trials have demonstrated inconsistent efficacy (5, 6), with a recent published meta-analysis by Scholte et al. showing favorable effect of home telemonitoring systems on all-cause mortality and HF-related hospitalizations (7). This encourages further research in this area. In the randomized, prospective AMULET trial, we revealed that outpatient telecare, based on nurse-led, non-invasive assessments supported by specialist teleconsultations, significantly improved clinical outcomes in patients after an episode of acute HF. During the 12-month follow-up, when compared to standard care, AMULET telecare reduced the risk of the first unplanned HF hospitalization (HR 0.62; *p* = 0.015) as well as the risk of total unplanned HF hospitalizations (HR 0.64, *p* = 0.044) (8).

The objective of this analysis was to assess the influence of the AMULET intervention on the quality of life and self-care of HF patients compared with standard care.

## 2 Materials and methods

### 2.1 Study population

The objectives and design of the AMULET study have been previously reported in detail (9). In brief, AMULET enrolled 605 patients aged 18 and older with HF, a left ventricular ejection fraction (LVEF) ≤ 49%, and at least one hospitalization due to acute HF decompensation (with clinical presentation of New York Heart Association [NYHA] functional class III–IV) in the 6 months prior to enrollment. Patients were randomly assigned to the telecare (*n* = 300) or standard care (*n* = 305) groups. The telecare group (AMULET intervention) was subject to regular ambulatory visits with patients’ clinical assessments and HF education performed by trained nurses; additional remote specialist teleconsultations were performed as needed. In the standard care group, patients were followed according to standard practices in the existing healthcare system.

Quality of life and self-care assessments were performed at baseline and at the 12-month follow-up visit. Each study participant provided written informed consent to participate in the study. The trial was approved by the local ethics committee (no. 70/WIM/2016). ClinicalTrials.gov Identifier: NCT03476590.

### 2.2 A new model of medical telecare: AMULET intervention

Seven regular outpatient visits according to a predefined schedule were performed by nurses at the ambulatory care point (ACP). Each visit included:

- Assessment of HF signs and symptoms.
- Noninvasive hemodynamic assessment (heart rate, systolic and diastolic blood pressure, total body water, thoracic fluid content using impedance cardiography and bioimpedance techniques).
- Presentation of the results via a web-based telemedicine service to a remotely available cardiologist.
- Providing details of the therapeutic decision to the patient.
- Individual adjustment of the scope of education depending on the patient’s needs.

Patient education was performed at recruitment for both groups and at each consecutive visit for the telecare group only. It focused on dealing with HF signs and symptoms, the natural course of HF, coping with the chronic course of the disease, prevention of deterioration, recommended physical activities, nutrition recommendations, compliance with medical treatment, general principles of self-assessment, and a healthy lifestyle. The patients were given educational booklets and self-reported diaries for monitoring blood pressure and weight management. To ensure reliable and comprehensive education, the nurses completed HF training and had a checklist of educational issues to cover during each visit. After each visit, patients received recommendations made in writing to the attending physician.

### 2.3 Quality of life assessment

Patient-reported quality of life was assessed using the 36-item Short Form Health Survey (SF-36), Minnesota Living with Heart Failure Questionnaire (MLwHF), and the European Heart Failure Self-care Behavior Scale (EHFScBS-12).

The SF-36 is one of the most widely used generic quality of life measurement tools and meets the required psychometric standards (10). The SF-36 questionnaire consists of 36 items divided into eight scales: physical functioning, role-physical, bodily pain, general health, vitality, social functioning, role-emotional, mental health, and two summary scores (the Physical [PCS] and the Mental Component [PCS] Summary scores). Higher scores reflect a better quality of life, with a scale ranging from 0 to 100. For ease of interpretation and comparison, data are presented normalized to a mean score of 50 according to Polish data (11).

The MLwHF is a self-administered HF-specific questionnaire comprising 21 items answered on a 6-point Likert scale, representing different degrees of HF’s impact on QoL from 0 (none) to 5 (very much). It provides a total score on a range of 0 to 105, with a higher score indicating poorer QoL (12). The MLwHF has been shown to be a powerful predictor of morbidity and mortality among HF patients (13).

### 2.4 Self-care assessment

The EHFScBS-12 questionnaire contains 12 statements concerning the self-care capabilities of HF patients (14). Three aspects of self-care behaviors are covered: compliance with regimens (i.e., weight control, restriction of fluids, sodium-restricted diet, and adherence to medications and flu vaccinations), contacting medical staff upon recognition of symptoms of decompensation (i.e., dyspnea, fatigue, edema, and weight gain), and adapting activities (i.e., having enough rest and adjusting physical activity). The responses to the statements described above are given on a five-point Likert scale ranging from 1 (I completely agree) to 5 (I do not agree at all). The overall score is calculated by aggregating the points from all statements and ranges from 12 to 60; the higher the score, the lower the patient’s self-care capability. A mean value of 1 or 2 on the Likert scale represents a high or satisfactory level of self-care (15).

### 2.5 Statistical analysis

Statistical analyses were performed using Statistica 12.0 (StatSoft, Inc., Tulsa, United States). The distribution and normality of the data were assessed via visual inspection and the Shapiro–Wilk test. Continuous variables were presented as mean ± standard deviation (SD) or median and interquartile range (IQR). The changes in selected variables were calculated as: d_X (delta) = absolute value at the end of follow-up—absolute value at baseline. For comparative analyses, the study group was stratified by allocation to the telecare and standard care groups. These groups were compared using the Mann–Whitney U test. For this analysis, we used the available data only from patients who satisfactorily completed both the baseline and follow-up tests (separately for each test). The differences between the values of selected continuous variables from baseline to the end of follow-up were compared using the Wilcoxon matched pairs test. A general linear model for repeated measures was used to evaluate the effect, and a *p*-value of < 0.05 was considered statistically significant.

## 3 Results

### 3.1 Clinical baseline characteristics of the study population

In the AMULET study group 79% of the subjects were male, the mean age was 67 ± 14 years and mean LVEF was 32 ± 15%. The heart failure functional status according to the NYHA was class I in 63 (11%) 166 subjects, class II in 390 (65%) subjects, and class III in 144 (24%) subjects. The comorbidities included: previous myocardial infarction (43%), previous stroke (10%), hypertension (61%), diabetes (39%), atrial fibrillation or flutter (55%). Additional demographic and medical characteristics of this patient population are presented in our previous paper (8). The AMULET intervention, reduced the risk of the primary endpoint (first unplanned HF hospitalization or cardiovascular death) during the 12-month follow-up by 31% (*p* = 0.044) (8).

### 3.2 Quality of life and self-care at baseline

Complete SF-36 data were available for 223 patients randomized to telecare and 211 patients randomized to standard care. For the overall study group, the median baseline PCS and MCS scores were 43.3 and 58.4, respectively. Complete MLwHF data were available for 74 patients randomized to telecare and 72 patients randomized to standard care. For the overall study group, the median baseline MLwHF score was 48.0. Complete EHFScBS data were available for 165 patients randomized to telecare and 155 patients randomized to standard care. For the overall study group, the median baseline EHFScBS score was 33.0. There were no statistically significant differences in the baseline scores between the telecare and standard care groups for all three questionnaires (Table 1).

**Table 1:** The intergroup comparison of baseline and follow-up values, as well respective changes for SF-36, MLwHF, and EHFScBS-12.

Variable	All Mean ± SD; mediana (IQR)	Standard care Mean ± SD; mediana (IQR)	Telecare mean ± SD; mediana (IQR)	*p*-value Telecare vs. standard care
**SF-36**				
PCS_baseline	43.9 ± 12.5; 43.3 (35.2–51.7)	44.1 ± 12.4; 43.4 (35.2–52.2)	43.7 ± 12.6; 43.3 (35.2–51.6)	0.860
PCS_follow-up	47.0 ± 12.9; 47.4 (38.6–55.5)	46.9 ± 12.8; 46.6 (38.6–55.1)	47.1 ± 13.1; 47.4 (38.2–55.9)	0.784
∆ PCS	3.1 ± 11.4; 2.0 (−3.4–9.2)	2.8 ± 10.0; 2.0 (−3.1–8.3)	3.43 ± 12.7; 2.0 (−3.5–9.5)	0.717
MCS_baseline	60.8 ± 21.4; 58.4 (46.8–74.0)	62.0 ± 20.2; 60.4 (46.8–76.0)	59.6 ± 22.4; 58.4 (44.8–74.0)	0.303
MCS_follow-up	63.9 ± 21.0; 63.3 (48.7–79.9)	64.5 ± 20.4; 64.3 (50.7–81.8)	63.4 ± 21.5; 62.3 (48.7–77.9)	0.471
∆ MCS	3.1 ± 20.8; 1.9 (−7.9–14.3)	2.5 ± 18.6; 0.6 (−7.3–11.7)	3.8 ± 22.7; 3.9 (−7.8–17.5)	0.245
**MLwHF**				
MLwHF_baseline	48.3 ± 24.7; 48.0 (31.0–70.0)	48.8 ± 22.5; 49.5 (33.0–65.0)	47.9 ± 26.8; 47.0 (25.0–73.0)	0.793
MLwHF_follow-up	35.2 ± 24.6; 34.5 (14.0–50.0)	36.7 ± 25.5; 35.0 (14.5–53.0)	33.8 ± 23.7; 29.0 (12.0–48.0)	0.557
∆ MLwHF	−13.1 ± 24.6; −8.0 (−27.0–0.0)	−12.2 ± 29.9; −8.0 (−18.5–0.0)	−14.1 ± 28.6; −7.5 (−35.0–0.0)	0.837
**EHFScBS-12**				
EHFScBS_baseline	32.6 ± 8.9; 33.0 (26.0–39.0)	32.2 ± 9.2; 32.0 (25.0–38.0)	33.1 ± 8.6; 34.0 (27.0–40.0)	0.198
EHFScBS_follow-up	30.3 ± 8.8; 30.0 (24.0–36.0)	31.3 ± 8.6; 32.0 (25.0–37.0)	29.6 ± 8.9; 29.0 (23.0–36.0)	0.080
∆ EHFScBS	−0.7 ± 21.8; 0.0 (−12.5–12.0)	−0.3 ± 21.6; 0.0 (−11.0–11.0)	−1.0 ± 22.0; 0.0 (14.0–14.0)	0.813

SF-36, Short Form 36 questionaire; MlwHF, Minnesota Living with Heart Failure Questionnaire; EHFScBS-12, European Heart Failure Self-care Behavior Scale; SD, standard deviation; IQR, interquartile range; PCS, physical component score; MCS, mental component score; ∆, delta.

### 3.3 Quality of life and self-care at the 12-month follow up

**Figure 1:**
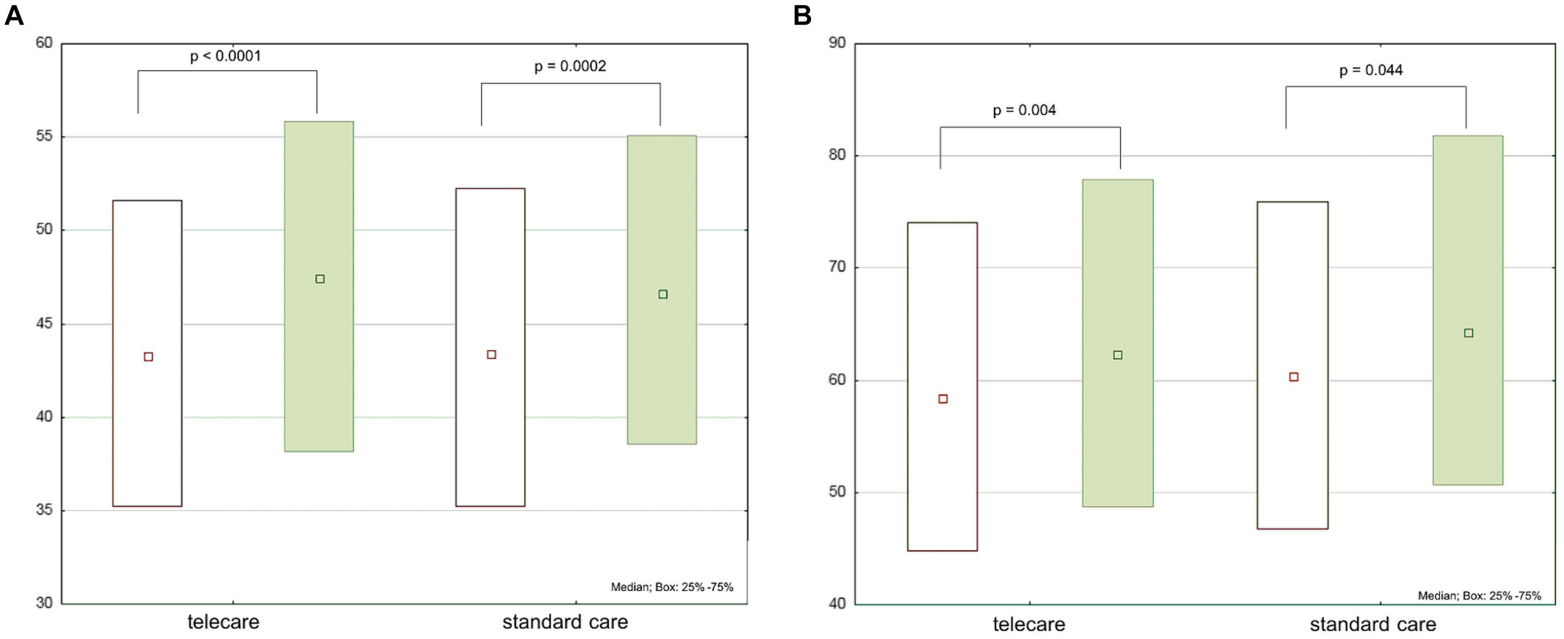
Physical **(A)** and mental **(B)** component scores (PCS and MCS, respectively) of the 36-item Short Form Health Survey (SF-36) scores at baseline (white boxes) and after 12 months (green boxes) by study group.

**Figure 2:**
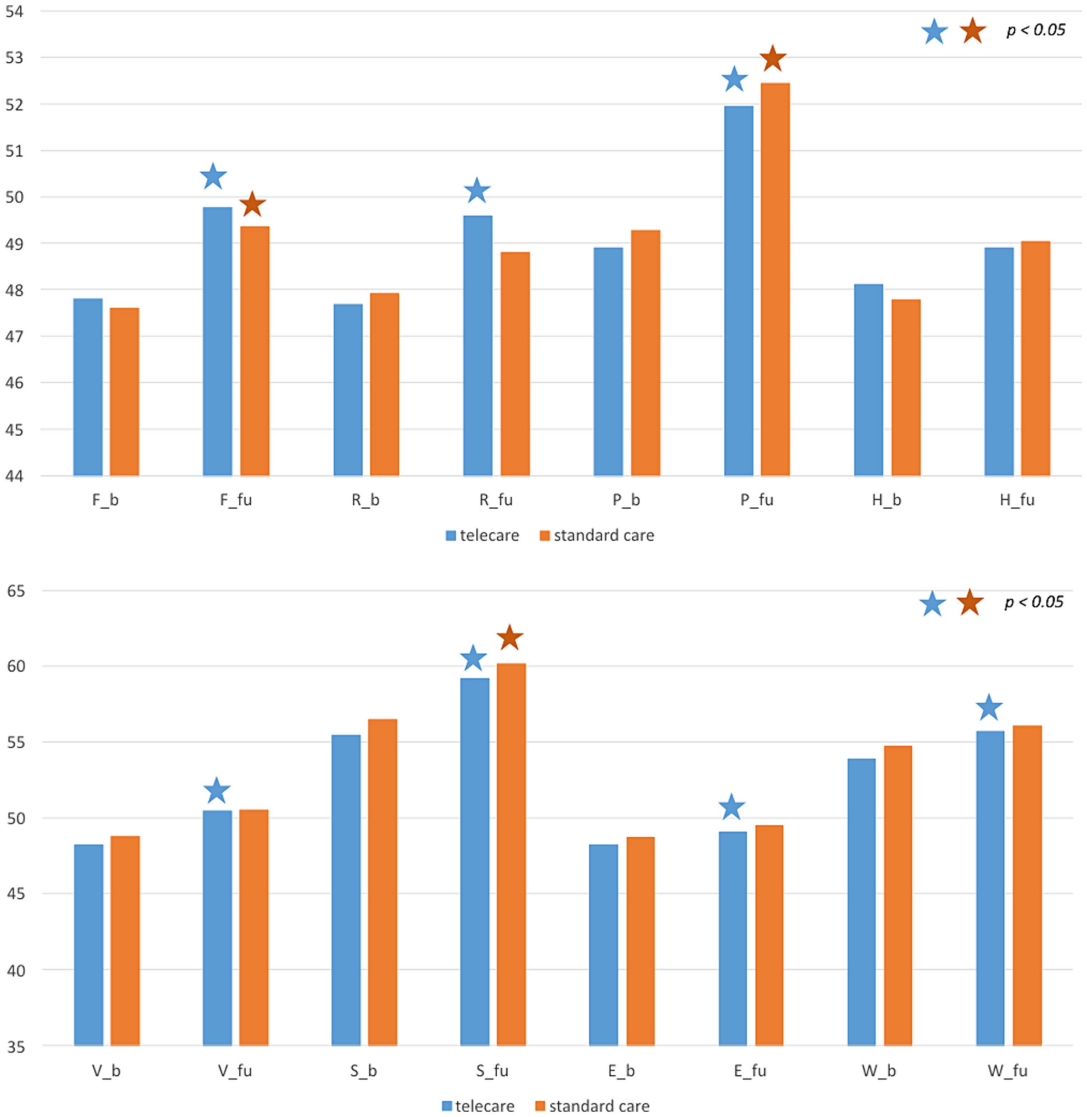
Specific domain scores at baseline and after 12 months by study group. F: physical functioning, R: role-physical, P: bodily pain, H: general health, V: vitality, S: social functioning, E: role-emotional, M: mental health, _b: baseline, _fu: follow-up.

At 12 months post-enrollment, statistically significant increases in PCS and MCS were noted, but with no intergroup differences (Table 1; Figure 1). Regarding specific domain scores, there were significant improvements in physical functioning, role-physical, and bodily pain in the telecare group. As for the standard care group, there were significant improvements in physical functioning and bodily pain. There were no significant changes in the general health domain in either group. Within the MCS, significant increases were noted in vitality, social functioning, role-emotional, and mental health in the telecare group, while the standard care group scored higher only in social functioning (Figure 2).

**Figure 3:**
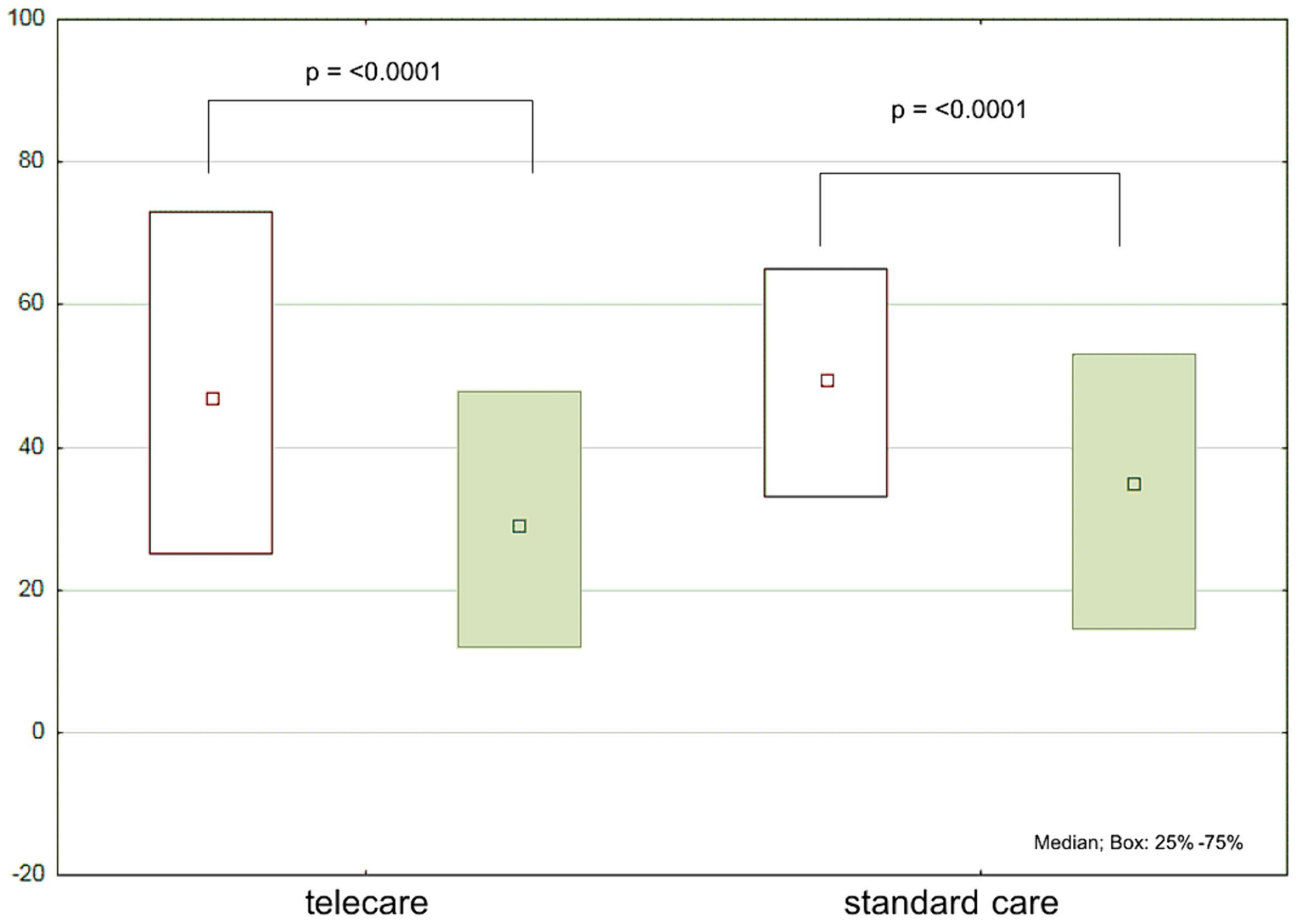
Minnesota Living with Heart Failure Questionnaire (MLwHF) scores at baseline (white boxes) and after 12 months (green boxes) by study group.

There was a significant decrease in MLwHF scores at follow-up in both groups. However, the difference between the groups did not reach statistical significance (Table 1; Figure 3).

**Figure 4:**
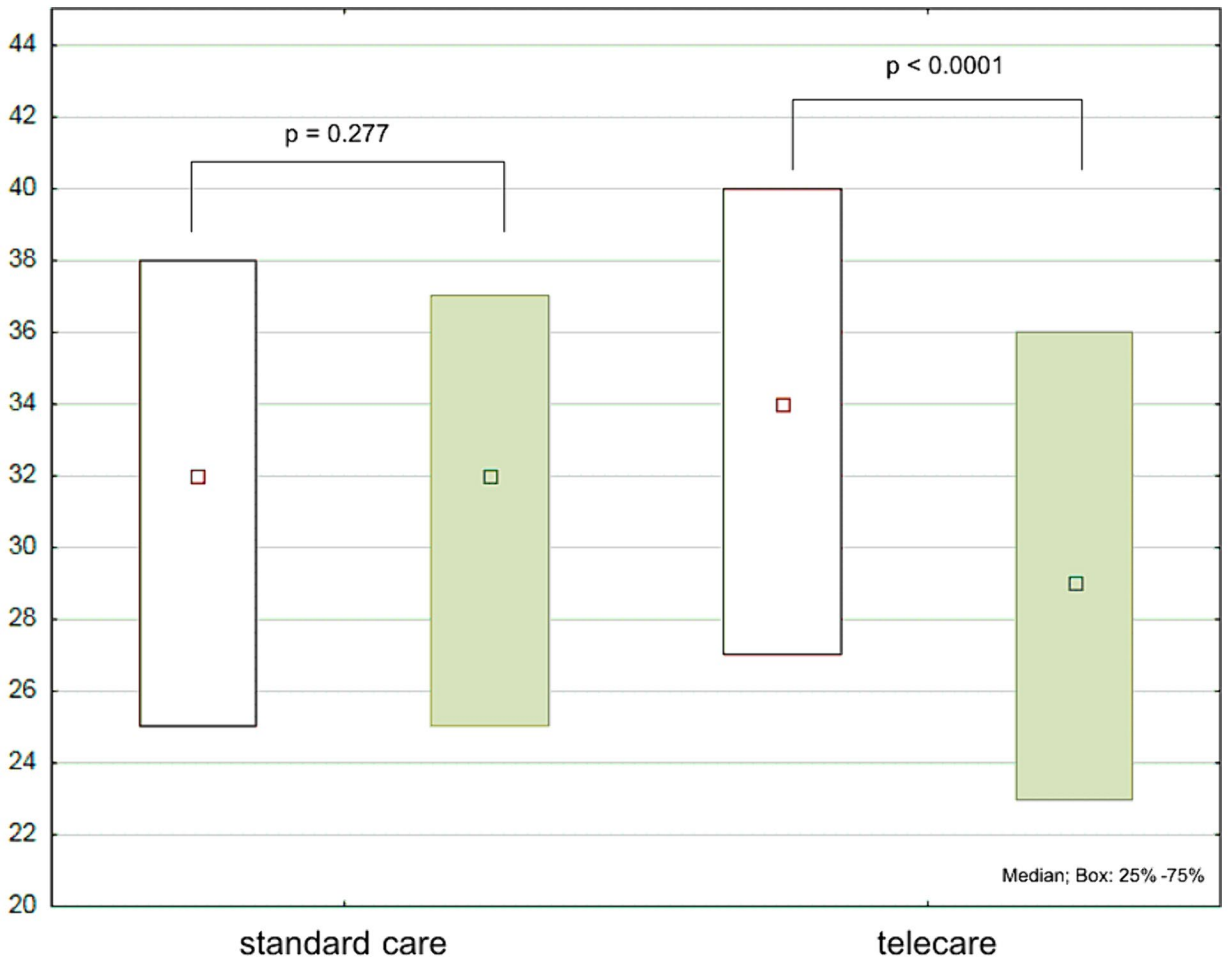
European Heart Failure Self-care Behavior Scale (EHFScBS-12) scores at baseline (white boxes) and after 12 months (green boxes) by study group.

Regarding the total EHFScBS-12 scores, there were no significant changes in the standard care group, while a significant decrease was noted in the telecare group (Figure 4). The analysis of individual items of the EHFScBS-12, which describe specific aspects of self-care behaviors, revealed that the beneficial effect of AMULET intervention in the telecare group was derived from improvements in adherence to everyday weight measurement, contact with medical staff in case of rapid weight gain or increased fatigue, reduced-salt diet, and increased exercise activity. While there were no intergroup differences at baseline, at the 12-month follow-up visit, telecare patients had better adherence to everyday weight measurement, limitation of fluid intake, and regular exercise than patients receiving standard care (Table 2).

**Table 2:** Scores for individual items of EHFScBS-12 questionnaire and the percentage of participants presetting with low levels of self-care by intervention at baseline and follow up.

	Low/unsatisfactory* level of self-care					
	Baseline			Follow-up		
	Standard care	Telecare	p (standard care vs. telecare)	Standard care	Telecare	p (standard care vs. telecare)
Item	*n* (%)	*n* (%)		*n* (%)	*n* (%)	
I weigh myself every day	80 (51.6)	91 (55.2)	0.529	85 (54.8)	62 (37.6)^#^	0.002
If I get short of breath I take it easy	35 (22.3)	46 (27.9)	0.276	38 (24.5)	48 (29.1)	0.356
If my shortness of breath increases I contact my doctor or nurse	80 (51.6)	94 (57.0)	0.336	85 (54.8)	85 (51.5)	0.552
If my feet/legs become more swollen than usual I contact my doctor or nurse	83 (53.6)	97 (58.8)	0.345	84 (54.2)	82 (49.7)	0.421
If I gain 2 kilo in 1 week I contact my doctor or nurse	100 (64.5)	112 (67.9)	0.525	98 (63.2)	91 (55.2)^#^	0.142
I limit the amount of fluids I drink (not more than 1½–2 L/day)	73 (47.1)	67 (40.6)	0.242	68 (43.9)	52 (31.5)	0.023
I take a rest during the day	30 (19.4)	43 (26.1)	0.153	32 (20.7)	44 (26.7)	0.206
If I experience increased fatigue I contact my doctor or nurse	103 (66.5)	115 (69.7)	0.533	99 (63.9)	98 (59.4)^#^	0.411
I eat a low salt diet	82 (52.9)	96 (58.2)	0.342	79 (51.0)	71 (43.0)^#^	0.155
I take my medication as prescribed	12 (7.7)	23 (13.9)	0.076	10 (6.5)	16 (9.7)^#^	0.288
I get a flu shot every year	111 (71.6)	112 (67.9)	0.468	105 (67.7)	105 (63.6)	0.440
I exercise regularly	123 (79.4)	130 (78.8)	0.901	123 (79.4)	114 (69.1)^#^	0.036

^*^3–5 on points on the Likert-type scale (from 1 – “I completely agree” to 5 - “I do not agree at all”).

^#p < 0.05 for change from baseline to follow-up.^

## 4 Discussion

In this study, we demonstrated that AMULET telecare positively influenced specific domains of quality of life and improved the self-care of HF patients.

The mean of the PCS of the SF-36 for the overall study group was 43.9, which is below the mean for a healthy population, but is consistent with the results of previous studies on patients with cardiovascular diseases (16), though one study reported an even lower mean PCS of 33.3 in HF patients (17). This poorer score relative to that of patients suffering from other chronic diseases stems from the typical signs and symptoms of HF, which strongly limit physical performance. The mean MCS of the SF-36 in our study was 60.7, which is above the mean for a healthy population (standardized mean of 50) (10). The mean age of the study population was 67 years. With this in mind, it can be concluded that older HF patients appear to have less mental distress because of the abundance of coping strategies available when facing diseases at a greater age.

**Figure 5:**
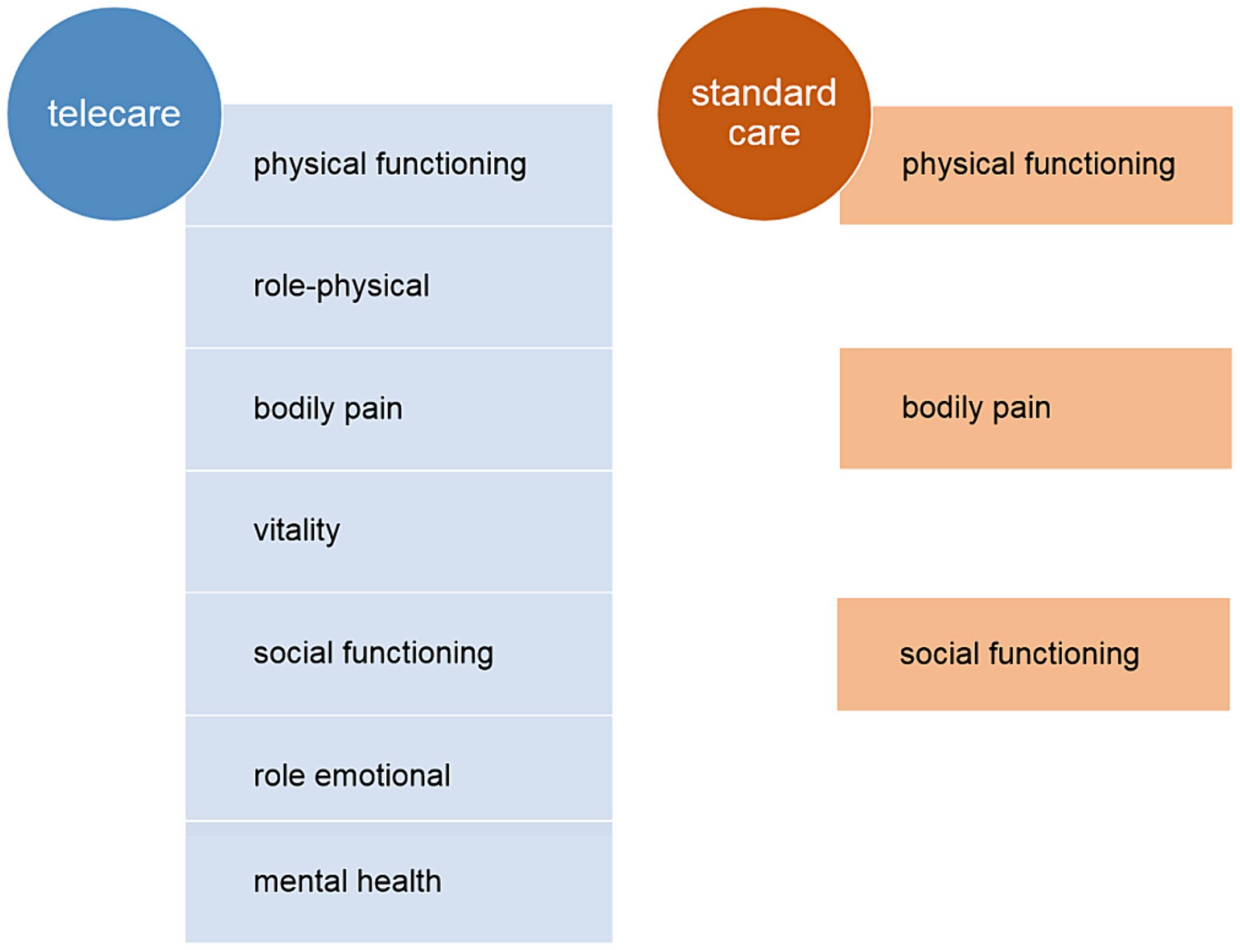
The quality-of-life-specific domains significantly improved within 12 months of follow-up under both telecare and standard care.

There were no significant differences between the telecare and standard care groups when the summary components of the SF-36 questionnaire, which assess the general level of HRQoL, were considered. To obtain more information regarding the specific needs of HF patients, we analyzed the specific SF-36 domains. In both groups, improvements in physical function, pain perception, and function in social relationships were observed. However, perceptions of overall health did not differ between the two groups and did not change significantly after 12 months. The new telecare model had a positive impact on reducing limitations in self-care, physical activity, social activity, and roles resulting from health problems. At the 12-month follow-up, subjects in the telecare group reported more positive affect, less psychological distress, and fewer limitations in usual social and role activities due to emotional problems when compared to the standard care group (Figure 5). Optimal care program and knowledge about the disease increases patient’s self-efficacy in dealing with symptoms and prevention decompensation. Improved personal coping, disease confidence positively affects patient’s feelings that is reflected by improvement in specific mental health domain (17). The limited effect of the AMULET intervention on SF-36 scores might be partly explained by the fact that the SF-36 is not specifically intended for patients with cardiovascular diseases and is less sensitive to changes in clinical status during or after interventions (17).

Analysis of the MLwHF questionnaires revealed positive changes in coping with HF, as evidenced by average decreases of 14 and 12 points in the telecare and standard care groups, respectively. However, no intergroup differences were noted for the MLwHF results. This improvement may have some important consequences for the patients’ prognosis. Alla et al. (18) analyzed the results of 108 patients registered in the EPICAL program (hospitalized patients with HF, NYHA grade III/IV, edema or hypotension, and LVEF <30%) and reported that a 10-point decrease from baseline in the MLwHF score was associated with a 23–36% increase in the risk of death or hospitalization for HF. It was also a predictive factor of survival and an independent predictive factor of hospital-free survival in patients with advanced HF.

The benefits of AMULET telecare were most obvious in the domain of self-care. Self-care comprises the process of establishing behaviors undertaken by the individual to ensure healthy functioning, holistic well-being, and the ability to cope actively with illness when it occurs (19). In HF, several behaviors positively contribute to the well-being of patients, namely fluid intake and weight gain control, adequate nutrition and exercise, adherence to medication, and recognition of symptoms that might lead to decompensation. In our study, we used a short and practical self-reported assessment covering three components of self-care behaviors related to coping with heart failure: regimen compliance, asking for help, and adapting activities. The mean total self-care score in the studied population was 32.6, indicating a significant deficit in self-care. When compared with standard care, AMULET telecare resulted in better adherence to everyday weight measurement, increased exercise activity, better dietary habits, and more frequent contact with medical staff in cases of symptoms suggestive of HF deterioration. The possible effects of improved self-care include substantially reduced adverse clinical outcomes and health care costs (20). The results of our study are consistent with those from the study by McAlister et al. (21), which demonstrated a beneficial effect on the reduction of hospitalization by implementing healthcare interventions with a focus on educational programs and the promotion of self-care. According to the authors of the Polish adaptation of the EHFScBS (22), the questionnaire can be used to assess the self-care capabilities of patients with HF, and its results can be an appreciated source of information on the effectiveness of educational activities. These are confirmed by the presented results of the AMULET trial.

Wiśnicka et al. (23) assessed the level of self-care and quality of life in a cross-sectional study involving 80 Polish men with HF. The results highlighted low levels of self-care, and the authors indicated the need for appropriate education to improve clinical outcomes. Educational interventions conducted by nurses, implemented as part of the AMULET project, meet these needs and significantly improve the level of self-care in patients with HF (23). Additionally, the results from the randomized TIM-HF2 trial confirmed the beneficial effect of remote patient management on self-care behavior in HF patients (24).

The AMULET model of nurse-led telecare corresponds to the current European Society of Cardiology (ESC) position paper recommendations regarding the self-care of HF patients (25). A practical approach of delivering HF care has been proposed based on the 3 fundamental concepts of self-care: maintenance, monitoring, and management. Self-care maintenance addresses behaviors to maintain clinical stability (e.g., nutritional status, optimal exercise, and adherence to medication). Self-care monitoring involves observing changes in signs and symptoms and can be performed by patients themselves (using a blood pressure and weight control diary and self-assessment with a visual analog scale), as well as by nurses at in-person visits. Self-care management involves responding to changing signs and symptoms, such as by adjusting dosing of diuretics and other medications or changing activity levels. In the AMULET study, both groups received patient education at the beginning of the study, and in the telecare group, it was consolidated at subsequent visits. Patient self-assessment was supplemented with examinations by a nurse, which involved noninvasive assessments that provided objective data on the patients’ hemodynamic status. The nurse-led in-person visit was supported by web-based decision-making by cardiologists. This complex evaluation and careful approach could positively influence the patient’s engagement in self-care. Patients with heart failure had multiple morbidities, the intervention included education and contact with a nurse, and the QoL effect that we assessed in this study could have been contributed to by a beneficial effect on non-cardiac diseases. However, the patients were not subjected to a detailed assessment in this respect.

It is worth mentioning that monitoring alone is not enough. Domenichini et al. (26) reported that even with the use of an implantable device to actively monitor parameters that predispose to the occurrence of HF exacerbation, monitoring *is useless in the absence of adequate response of medical staff to system generated alerts/indication*. In the context of HF management, these results suggest a need for control by the medical team.

It has been previously documented that the AMULET intervention reduced the risk of the composite endpoint of cardiovascular death or first unplanned HF hospitalization by 31% (8). The results presented in the current paper complement and complete these clinically relevant results with evidence of their beneficial effects on patients’ self-reported quality of life and self-care. At every stage of HF management, improvements in disease-specific quality of life and self-care should be considered when assessing and optimizing the effects of treatment. The interaction of well-defined components of healthcare, such as transfer of care to outpatient settings, systematic education oriented to individual needs, telemanagement of clinical status, and compliance with non-pharmacological recommendations, has been shown to be effective in realizing these specific aims. It is worth mentioning that AMULET intervention met the recommendations to close follow-up of HF patients after discharge overcoming the shortage of cardiologists. However, still professional stuff (trained nurses) and modern technologies are essential in this model. It is also worth wondering how to provide AMULET intervention to patients with travel limitation (home-bound/frail patients). Home visits with remote set of measuring devices should be consider. Such technology was tested by the authors (27).

The main limitation of this study was missing data. This was mostly due to loss to follow-up as a result of difficulty in attending in-person follow-up visits due to the coronavirus disease 2019 (COVID-19) pandemic. It is preferable for questionnaires to be administered face-to-face in the presence of a trained interviewer. In psychological research, a return rate of 50–60% is expected, which should be considered when referring to our study. The three questionnaires were answered by a different number of patients which may lead to some discrepancies. The uneven number of completed tests might have resulted from the features associated with the use of self-reported questionnaires, where a large number of questions with overlapping topics discourages completion of the task. Furthermore, the questionnaires were completed during the pandemic where the presence of third parties and any supervision were not possible. All of the above may potentially influence the homogeneity of the results and should be considered while its interpretation. Moreover, the patients were enrolled within 6 months after hospitalization due to acute HF decompensation, which limited diversity in the course of HF and could influence the patients’ perception of quality of life. It should also be considered that the results at 12 months were limited to data available from people who attended the follow-up visit in person. Taking into account the COVID-19 pandemic, this could be a form of sampling bias. Subjects who show up to the follow-up visit are more likely to be in better health than subjects who were lost to follow up. This may partly explain the improvement in quality of life in both groups.

## 5 Conclusion

AMULET outpatient telecare, which is based on nurse-led, non-invasive assessments supported by specialist teleconsultations, improved the quality of life and self-care of HF patients after an episode of acute HF. This beneficial effect was significantly greater than in standard care in the domains of positive affect, psychological distress, limitations in usual social and role activities due to emotional problems, everyday weight measurement, increased exercise activity, better dietary habits, and more frequent contact with medical staff if patients develop symptoms suggestive of HF deterioration.

## Data Availability

The raw data supporting the conclusions of this article will be made available by the authors, without undue reservation.
